# Muriate of Ammonia in Syphilis

**Published:** 1869-01

**Authors:** Theodore Griffin

**Affiliations:** Chicago


ARTICLE III.

### MURIATE OF AMMONIA IN SYPHILIS.

BY THEODORE GRIFFIN, M.D., Chicago.

Being desirous to call attention to the efficacy of muriate of ammonia in secondary syphilis, I will narrate briefly the result of my experience with this article in two cases. I am satisfied that it is a valuable remedy, especially in the secondary form form of this disease. This conviction results from its successful employment where the usual remedies, combined and uncombined, failed.

Mr. ——— has for the past four years been the victim to secondary syphilis; the eruption being of the tubercular variety, and mostly confined to the lumbar region. Many of the sores being large and prominent, others excavated and ulcerating.

General health depraved. Sallow appearance of the skin. Pulse feeble, 90 per minute; indigestion. Mental depression, with conviction that he was diseased beyond the possibility of cure.

He was put upon the use of tonics and

℞. Iodide Potass., ---------------------- ʒij.

Bi. Chlo. Hy., ----------------------- gr. 1.

Mucilage Acaciæ, ------------------ ℥ij.

Ft. Sol. Liq. Take two teaspoonsful three times a day after meals.

This treatment, with warm baths and suitable hygienic measures, was strictly adhered to for two months, without any appreciable mitigation of the symptoms. Iodide of potass. and iodine, after Prof. Gunn's method (of Rush College), was tried; also iodide of fe., Stillingia, etc., almost *ad infinitum*, without avail.

Finally, the following combination was administered:—

℞. Iodide Potass., ---------------------- ʒij.

Bi. Chlo. Hy., ----------------------- gr. 1.

*Muriate Ammonia*, ------------------ ʒiij.

Mucilage Acaciæ, ------------------ ℥ij.

Ft. Sd. Liq. Take teaspoonful three times a day, after meals.

Within one week after the commencement of this mixture improvement began, and at the end of six weeks the tubercles were cleared off, and the ulcerations healed. The patient now considers himself cured.

CASE 2. Mr. ———, aged 22 years, unmarried, of intemperate habits. Contracted syphilis twelve years ago. One year following the disappearance of the primary symptoms, secondary symptoms appeared, which were subdued by his physician. For the past five years, until one year previous to his application to me, he had no manifestations of disease. During the past year he has been constantly under treatment for a papular eruption upon the breast, back, and legs. Hair-follicles diseased, with consequent loss of hair and eyebrows. Has been but little benefited by treatment. I put him at once upon the following:—

℞. Bi. Chlo. Hy., ---------------------- gr. ij.

*Muriate Ammonia*, ------------------ ℨss.

Mucilage Acaciæ, ------------------- ℥ij.

Ft. Sol. Liq. Take teaspoonful three times a day, after meals.

General tonics were dispensed with. Improvement began at once, and the man now considers himself well.

I am convinced that the cure in these cases cannot be attributed to the iodide potass. and bi. chlo. hy. used in these cases, for they had been tried faithfully in a variety of ways, without avail. Neither to the *vis med. naturæ;* but to the muriate of ammonia.

Dr. Durkee, of Boston, in his book on Gonorrhœa and Syphilis, alludes to the use of this article in syphilis. In conversation with him two years ago, he said, "I believe muriate of ammonia to be a remedy of value, and would like to have it extensively tried." The Germans entertain a high opinion of its alterative and resolvent properties, and consider that its action on the system closely resembles that of mercury: increasing the secretions, but improving the tone of the nervous system. Yet but little attention has been bestowed upon it as a remedy for syphilis, although its therapeutic properties indicate its use.

